# Cellular and immune landscape of chronic liver diseases: insights from immunophenotyping

**DOI:** 10.3389/fmolb.2024.1521811

**Published:** 2025-01-29

**Authors:** Shaghayegh Soleimani, Ozgur Albayrak, Kayra Somay, Hong Yang, Buket Yigit, Burge Ulukan, Dila Atak, Murat Akyildiz, Metehan Gursoy, Elif Demirtas, Adil Mardinoglu, Atay Vural, Murat Dayangac, Mujdat Zeybel

**Affiliations:** ^1^ Koç University Research Center for Translational Medicine (KUTTAM), Koç University, Istanbul, Türkiye; ^2^ Department of Gastroenterology and Hepatology, School of Medicine, Koç University, Istanbul, Türkiye; ^3^ Department of Internal Medicine, School of Medicine, Koç University, Istanbul, Türkiye; ^4^ Science for Life Laboratory, KTH - Royal Institute of Technology, Stockholm, Sweden; ^5^ Centre for Host-Microbiome Interactions, Faculty of Dentistry, Oral and Craniofacial Sciences, King’s College London, London, United Kingdom; ^6^ Department of Neurology, School of Medicine, Koc University, Istanbul, Türkiye; ^7^ Department of General Surgery, Medipol University, Istanbul, Türkiye

**Keywords:** chronic liver disease, hepatitis, liver immunophenotyping, hepatic inflammation, fibrosis

## Abstract

**Background:**

Chronic liver disease due to alcohol-related liver disease and chronic viral hepatitis pose a substantial burden on healthcare systems. Chronic liver disease may predispose to hepatocellular carcinoma, for which therapeutic options are limited. This study aimed to explore the immune cell characteristics of the clinical conditions.

**Methods:**

Explant liver samples were collected from 25 patients for bulk RNA sequencing and flow cytometry analysis. Immune cell populations were characterized by flow cytometry from isolated hepatic and peripheral mononuclear cells.

**Results:**

Significant differences in immune cell characteristics were observed among patients with three clinical conditions. Viral hepatitis and peri-tumor samples exhibited higher hepatic B cell counts compared to alcohol-related liver disease. Additionally, chronic liver disease patients showed higher levels of CD57^+^ T cells, suggestive of T cell differentiation. Differential expression analysis identified several genes associated with immune regulation, including downregulation of *CD27* and upregulation of *granzyme B* in ARLD, consistent with a highly differentiated phenotype. *LAG3* and *PDCD1* were upregulated in peri-tumor samples. The NK cell count was lower in peri-tumor liver specimens compared to ARLD, and an upregulation of *TIGIT*, an inhibitory marker, was observed in those peri-tumor specimens.

**Conclusion:**

This study contributes to the understanding of immune dynamics in chronic liver disease among different etiologies. B lymphocytes are relatively reduced in alcohol-related liver disease compared to other groups, and T cells exhibit a more differentiated subtype. The peritumor microenvironment in HCC suggests a relatively diminished presence of NK cells and a potential tendency toward increased inhibitory characteristics.

## 1 Introduction

Chronic liver disease (CLD) represents a significant challenge to global health, characterized by persistent liver inflammation and fibrosis. It exacts a heavy toll, claiming over 1 million deaths annually worldwide and ranks among the leading causes of mortality in developed countries (Thomas, 2019; Huang et al., 2023). The main etiologies contributing to CLD include chronic viral infections, alcohol misuse and metabolic dysfunction-associated steatotic liver disease (Mehta et al., 2020). Viral hepatitis, chronic inflammation and aberrant wound-healing response pose an increased risk of hepatocellular carcinoma (HCC), the most common form of primary liver cancer.

Chronic hepatic injury leads to persistent necro-inflammation and a gradual buildup of fibrosis, a process driven by the activation of hepatic stellate cells (Ebrahimi et al., 2016). The histopathologic pattern of fibrosis varies according to etiology. For example, chronic viral hepatitis (CVH) often causes fibrosis around the portal tracts, with severe cases resulting in bridging fibrosis (Ebrahimi et al., 2016). Alcohol-related liver disease (ARLD), in particular, is associated with its unique abnormalities, such as increased hepatic lipid deposition and intestinal permeability, facilitating the transport of microbial toxins from the gut to the liver. These processes trigger an immune response that can lead to hepatic damage, fat accumulation, and fibrosis, initially starting from the pericellular area and advancing to portal/periportal fibrosis (An International Group, 1981).

CLD increases the risk of hepatocellular carcinoma (HCC). Specifically in hepatitis B, the risk escalates with the integration of viral DNA into the host genome, destabilizing it and prompting mutations in critical cancer-suppressing genes such as p53 and the WNT/β-catenin pathway (Levrero and Zucman-Rossi, 2016; Villanueva, 2019). ARLD involves complex mechanisms, including those directly related to alcohol metabolism, oxidative stress, DNA methylation, and abnormal iron and retinoid metabolism, which together foster a microenvironment for carcinogenesis (Ganne-Carrié et al., 2018; Sidharthan and Kottilil, 2014). Carcinogenesis in the liver is critically influenced by the immune response. The liver, densely populated with innate immune cells, such as macrophages and innate lymphoid cells, plays a pivotal role in maintaining the balance between immunity and tolerance, especially in hepatotropic virus infections. The capability of the liver to switch from immune tolerance to active immunity in response to infection or damage is critical. CVH or ARLD can activate the immune cells in different mechanisms. The recruitment and local activation of various immune cells, especially T and B lymphocytes along with NK cells, followed by the activation of hepatic stellate and macrophages, can lead to either resolution or progression of liver disease (Heymann and Tacke, 2016).

In this study, we analyzed circulating and hepatic immune cell dynamics of CLD due to viral and alcohol-related injuries and peritumoral (HCC) parenchymal diseases. We further explored hepatic transcriptomics in viral hepatitis, alcohol-related and peri-tumor specimens to highlight immune differences in CLD, which have improved our understanding of how the hepatic immune environment undergoes reprogramming in different injuries. This study, which integrates hepatic bulk RNA-seq data with blood and liver flow cytometry analysis, offers a comprehensive view of immune alterations in CLD.

## 2 Materials and methods

### 2.1 Patient recruitment

The use of human tissue and biological samples was approved by Koç University Ethics Committee with the approval number 2016.024.IRB2.005 and 2017.139.IRB2.048. Patients were recognized by specialists at Medipol and Florence Nightingale Hospitals, Istanbul, Turkey, and samples were collected following patients’ written consents. Liver specimens were collected from explant livers, and peri-tumor samples were obtained from a distance of at least 2 cm from the tumor. Histopathological scoring was performed according to the Ishak pathological staging. Patients with hepatocellular carcinoma along with viral hepatitis infection, alcohol-related liver disease, and viral hepatitis were included in this study, while other concurrent or alternate liver diseases (Primary Biliary Cholangitis, Primary Sclerosing Cholangitis, hemochromatosis, α1-antitrypsin deficiency, Wilson’s disease, or autoimmune liver disease) were excluded. Patients who had loco-regional treatments such as TACE or SIRT within the 6 months were not included in this study. Male and female alcohol consumers whose daily uptake was more than 60 g and 40 g, respectively, were included in the Alcohol-related liver disease cohort. BCLC stage and FIB-4 scores were assessed accordingly. All laboratory test results, in addition to other relevant clinical details such as gender, age, and etiology, were collected at the time of liver transplantation and phlebotomy.

### 2.2 Hepatic and peripheral blood mononuclear cell isolation

The mononuclear cells were isolated from explanted livers by a non-enzymatic process and using the Ficoll gradient. Liver tissues were minced into smaller pieces and added to the stomacher bag along with serum-free RPMI-1640 and subjected to incubation in the stomacher machine. After the incubation, the mixture was passed through a 125-pore size nylon mesh. Following the washing steps, the final pellet was dissolved in 1X DPBS, gently layered onto the Ficoll solution (density 1.077 g/mL), and centrifugation was performed. A washing step was applied to the collected cells to eliminate any residual Ficoll. The pellet was dissolved in freezing media containing 90% Fetal Bovine Serum (FBS) and 10% Dimethyl Sulfoxide (DMSO) for cryopreservation.

Peripheral blood mononuclear cell isolation procedure involved the addition of DPBS to an equal volume of collected whole blood. The blood and DPBS mixture was gently poured onto the Ficoll solution with a density of 1.077 g/mL at a slight angle, allowing for the distinct layering of components. Following centrifugation steps, the buffy coat containing mononuclear cells was collected. The pellet was dissolved in 1X DPBS, and freezing media (90% Fetal Bovine Serum (FBS) and 10% Dimethyl Sulfoxide (DMSO)) was added to the remaining cells for storage in liquid nitrogen.

### 2.3 Surface immunophenotyping

Mononuclear cells isolated from hepatic and peripheral blood samples of patients were seeded into a 96-well U-bottom plate with 100 μL of 1X DPBS, followed by the addition of Zombie NIR viability dye (Biolegend, United States). Following the incubation on ice for 10 min, the plate was centrifuged at 500 ×g for 5 min, and after discarding the supernatant, 100 μL of staining buffer (1X DPBS +1% BSA) containing the antibody was added to each well and incubated in the dark on ice for 20 min. The panel of antibodies used for surface immune phenotyping is detailed in Supplementary Table S1.

Following centrifugation, the supernatant was discarded, and the samples were resuspended in 200 μL of staining buffer before being transferred to 12 × 75 mm Falcon tubes. The samples were analyzed using the Attune NxT acoustic flow cytometer (Thermo Scientific, United States). FlowJo v10.8.1 software (BD Biosciences, United States) was used for flow cytometry data analysis.

### 2.4 RNA extraction and transcriptome sequencing

For total RNA extraction, snap-frozen human liver tissues were subjected to homogenization using stain-free steel beads in PowerLyzer 24 (Hilden, Germany) followed by Zymo Research Quick RNA Miniprep kit (Irvine, CA, United States) manufacturer’s protocol. The quality and quantity of the extracted RNA were assessed spectrophotometrically using NanoDrop (Thermofisher, Waltham, MA, United States), and the RNA integrity number (RIN) values were measured by TapeStation (Agilent Tech, Santa Clara, CA, United States). The amount of total RNA was measured more accurately by fluorometric determination with an RNA Broad Range kit (Thermofisher, Waltham, MA, United States) by using Qubit (Thermofisher, Waltham, MA, United States). Preparation of total RNA-Seq libraries was performed with Illumina Stranded Total RNA Prep and Ligation with Ribo-Zero Plus kit following the manufacturer’s standard protocol. The libraries were then pair-end (2 × 100 bp) sequenced on the NovaSeq 6000 platform, generating, on average, 25 million fragment reads per sample. Raw sequence data (.bcl) was converted to FASTQ with the Dragen Bio-IT platform (v3.9.5). The quality of RNA-seq data was assessed by FastQC (v0.11.9). Detailed information about the reagents and kits is provided in Supplementary Table S2.

### 2.5 RNA-seq data processing and differential expression analysis

Bulk RNA-seq data from tissues were aligned and quantified using Kallisto (v0.46.2) following a standard procedure against the Human genome (GRCh38, version 102) downloaded from Ensembl official website (https://www.ensembl.org/index.html). The output of Kallisto, including estimated counts and TPM (transcript per kilobase million)-based transcript-level expressions, were then converted to gene-level expressions by the Bioconductor package tximport (v1.22.0) with the tx2gene option set to link transcripts to the respective genes. Protein-coding genes were considered for the mentioned step and subsequent analyses. Differential expression analysis was carried out using the DESeq2 Bioconductor package (v1.34.0), following a standard protocol across all the pairwise comparisons.

### 2.6 Cell-type deconvolution using DWLS

Using the dampened weighted least squares (DWLS) algorithm on bulk tissue RNA-seq data, we estimated cell proportions. Cell type-specific transcription profiles and signature genes were employed via DWLS to estimate the proportions of various cell types. Liver fibrosis single-cell transcriptional data, previously generated by Ramachandran et al. (2019) (GSE136103), was used through the website presented by Duan et al. (2021). The FindAllMarkers function in the Seurat R package (v4.3.0) was used to identify cell-type specific genes. The transcript-per-million (TPM) values for marker genes from hepatic samples, combined with the signature reference, were input into DWLS to determine cell proportions.

### 2.7 Statistical analysis

Statistical analysis was performed using GraphPad Prism software version 9.5.1 (CA, United States). Mean ± SD was used for continuous normally distributed variables. The distribution of categorical variables between groups was determined using the χ2 test. Shapiro-Wilk test was conducted to assess the normality of the dataset. To assess variations between groups, One-Way ANOVA with Tukey’s correction for normally distributed variables and Kruskal-Wallis with Dunn’s correction for non-normally distributed variables were employed. All statistical significance was determined at a threshold of *p*-value < 0.05.

## 3 Results

**FIGURE 1:**
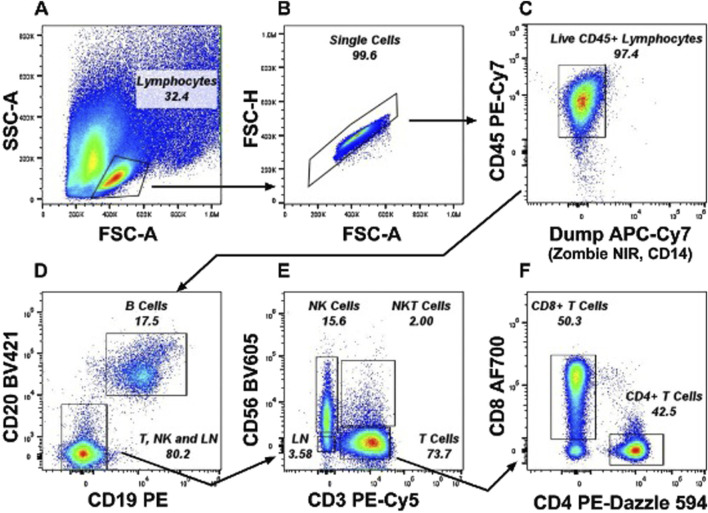
Representative gating strategy from a PBMC sample for Immunophenotyping experiments. Lineage cells (LN) were gated as CD56^−^ CD19^−^ CD14^−^ and CD3^−^ cells which excludes monocytes, B cells, NK cells and T cells and includes innate lymphoid cell population. **(A)** Lymphocytes were gated from the FSC-A x SSC-A plot. **(B)** Single Cells were selected from the FSC-A x FSC-H plot. **(C)** Alive CD45^+^ Lymphocytes were gated by excluding Zombie NIR^+^ dead CD14^+^ Monocytes. **(D)** B cells were selected as CD19^+^ CD20 bright cells and negative cells including CD20dim cells were gated as T, NK, and LN cells. **(E)** T cells, NK cells, NKT cells were gated as CD3^+^, CD56^+^ and CD3^+^CD56^+^ respectively. **(F)** CD4^**+**^ T Cells and CD8^+^ T Cells were gated from CD3^+^ T cells gate.

To evaluate the immune characteristics of hepatic and peripheral blood mononuclear cells in alcohol-related liver disease, chronic viral hepatitis, and hepatocellular carcinoma, we performed flow cytometry-based immunophenotyping in hepatic and peri-tumor explant specimens along with circulating leukocytes. We further performed bulk RNA sequencing (RNA-seq) gene expression profiling, particularly focusing on immunoregulatory pathway genes, in hepatic and peri-tumor parenchymal specimens. The study population consisted of 25 subjects who underwent liver transplantation due to ARLD (n = 7), CVH (n = 10) and viral hepatitis-associated HCC (n = 8). The detailed clinical data and patient characteristics of the cohorts are shown in Table 1. The gating strategy regarding flow cytometry is provided in Figure 1.

**TABLE 1:** A summary of clinical data of the cohorts.

Clinical characteristics	Viral hepatitis n = 10	Viral hepatitis and hepatocellular carcinoma n = 8	Alcohol-related liver disease n = 7	*p*-value
Age (Mean ± Sd)	49 ± 6	60 ± 5	58 ± 9	0.0143* H
Gender (F/M)	1/9	0/8	0/7	0.4578 †
Diabetes (Yes/No)	3/7	2/6	3/4	0.7491 †
ALT (IU/L)	55.1 ± 60.36	44.89 ± 31.54	22.86 ± 15.04	0.1611 H
AST (IU/L)	76.9 ± 79.15	48.13 ± 32.08	38.86 ± 23.02	0.4604 H
Albumin (g/dL)	29.70 ± 5.85	37.99 ± 4.75	30.93 ± 2.89	0.0041** F
Platelets (×10^9)	67.8 ± 35.05	101.0 ± 58.46	104.3 ± 49.41	0.205 H
FIB-4 Index	8.95 ± 6.06	5.13 ± 2.30	5.23 ± 2.484	0.1212 F
Fibrosis Stage (F0/F1/F2/F3/F4)	0/0/2/0/8	0/1/0/0/7	0/0/0/0/7	—
BCLC Stage (0/A/B/C/D)	—	1/3/4/0/0	—	—

Data are expressed as mean ± SD. For categorical variables’ distribution between groups, χ2 test was used. Shapiro-Wilk test was conducted to assess the normality. For normally and non-normally distributed variables among three groups, One Way ANOVA with Tukey’s Correction and Kruskal Wallis with Dunn’s Correction were performed, respectively. *p ≤ 0.05 and **p ≤ 0.01. † Χ2 test, H Kruskal Wallis test and F One Way ANOVA test.

**FIGURE 2:**
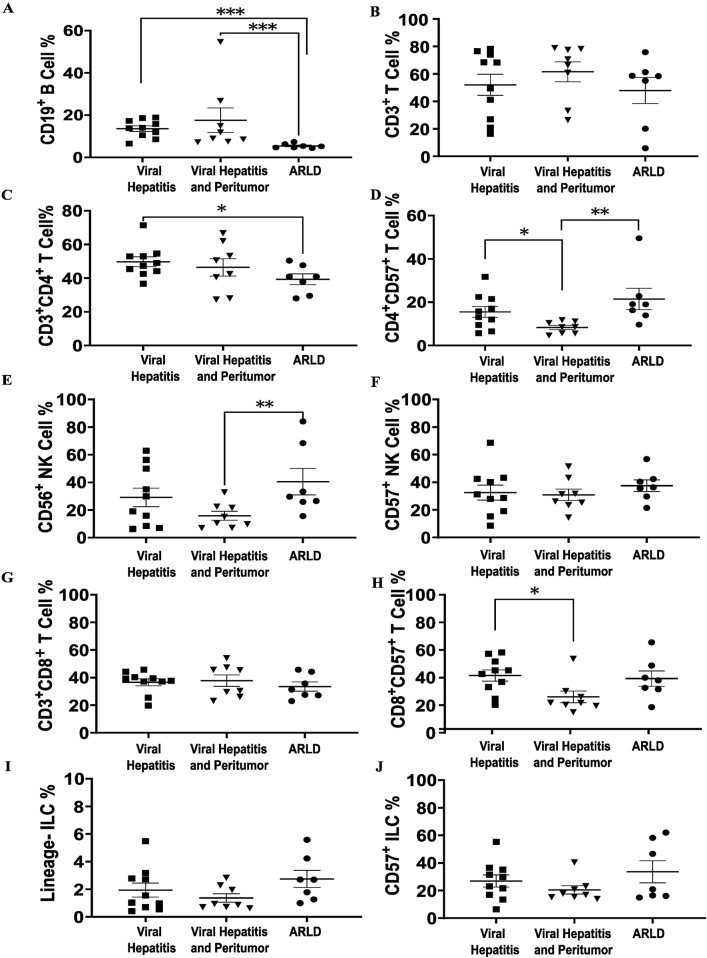
Liver immunophenotyping of different cohorts. Shapiro-Wilk test was used for the normality assessment. Non-normally distributed variables were assessed with Kruskal-Wallis with Dunn’s Correction. *p < 0.05, **p < 0.01 and ***p < 0.001. **(A, B)** Total liver B and T lymphocytes of three etiologies. B lymphocyte ratio is significantly lower in ARLD comparing to the other two groups, which can be as a consequence of alcohol consumption that may indicate alcohol dysregulation impact on humoral immunity. **(C)** Total CD4^+^ T cells is higher in viral hepatitis comparing to ARLD due to viral infection. **(D)** A rise is shown in CD57^+^ CD4^+^ T cells in ARLD and CVH which may indicate a senescence signal can elevate expression of granzymes. **(E)** CD56^+^ natural killer cell population is elevated in ARLD. **(F)** No significant difference was observed in CD57^+^ NK cells among all three population. **(G, H)** CD3^+^CD8^+^ and CD8^+^CD57^+^ T cell ratios in three groups, with an increase in CVH. **(I, J)** Hepatic lineage and CD57^+^ innate lymphocyte proportions in the three etiologies showed no substantial difference.

Flow cytometry-based analysis demonstrated that hepatic NK, T and B cells constituted the range of 18%–42%, 55%–64%, and 8%–20%, respectively, in the three cohorts. Hepatic B cells were significantly lower (*p* < 0.001) in ARLD compared to CVH and peri-tumor tissues (Figure 2A). While total hepatic T cell populations showed no substantial differences among the groups (Figure 2B), T cell subgroup analyses indicated lower levels of total CD4^+^ T cells in ARLD compared to CVH (Figure 2C). Moreover, CD4^+^CD57^+^ T cells were higher within the CVH and ARLD groups compared to peri-tumor tissues (Figure 2D). The percentages of CD56^+^ NK cells were lower in peri-tumor liver specimens than those in ARLD, whereas no significant differences were detected for CD57^+^ NK cells (Figures 2E, F). Total hepatic CD8^+^ T cells were similar in all groups; however, most strikingly CD8^+^CD57^+^ T cells were higher in CVH compared to peri-tumor tissues (Figures 2G, H). In addition, no significant differences were observed in hepatic innate lymphocyte populations across all groups (Figures 2I, J).

Flow cytometry-based circulating lymphocyte analysis demonstrated that NK cells, T cells, and B cells constituted the range of 22%–24%, 50%–55%, and 16%–20%, respectively, in blood samples across the three cohorts. These results revealed an approximate consistent distribution of CD19^+^ B cells, CD3^+^ T cells, and CD3^+^CD4^+^ T cells with no significant differences across the cohorts (Supplementary Figures S1A–C). While there was not any notable difference in CD57^+^ CD4^+^ T cells among those groups (Supplementary Figure S1D), the percentage of CD3^+^CD8^+^ T cells was lower in ARLD patients compared to viral hepatitis with HCC (Supplementary Figure S1E). Similar to CD57^+^ CD8^+^ T cells, this data showed a stable proportion of CD56^+^ and CD57^+^ NK cells and innate lymphocyte populations, suggesting no substantial variation in the peripheral innate immune cell population among all three etiologies (Supplementary Figures S1 F–J).

**FIGURE 3:**
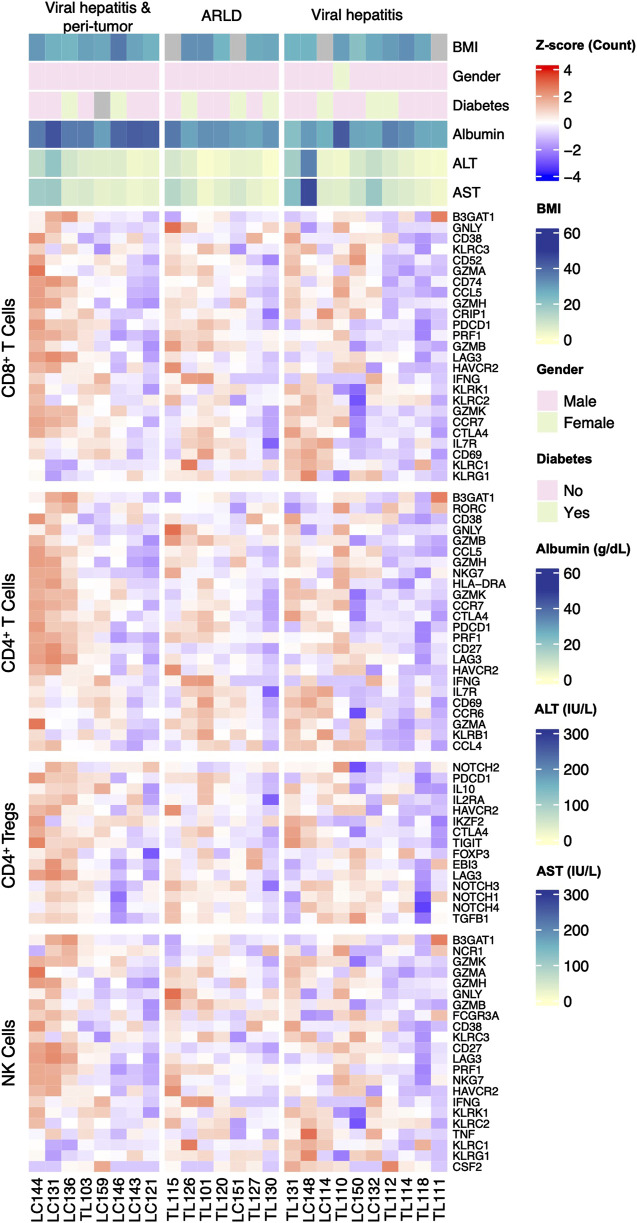
Expression pattern of the classical marker genes of each lymphocyte-subpopulation and clinical parameters in patients of all three groups. Lymphocyte-subpopulation markers expression was analyzed by bulk RNA sequence for all patients within ARLD, Viral Hepatitis, and viral hepatitis along with HCC groups. Related markers were categorized for cytotoxic T, helper T, regulatory T and natural killer cells.

The hepatic gene expression profiling through bulk RNA-seq was performed to explore lymphocyte-specific markers in three groups. Lymphocyte subpopulation-specific gene list is illustrated in Supplementary Table S3 and the related RNA sequence data is shown in Supplementary Table S4 for transparency, and the representative genes of these populations were presented in a heatmap (Figure 3). The RNA-seq analysis revealed five differentially expressed genes between the groups. *KLRC1* was downregulated in HCC compared to CVH (*p* = 0.03) and ARLD (*p* = 0.04). *CD27* showed significant downregulation in ARLD compared to CVH (*p* = 0.01) and HCC (*p* = 0.0002). *GZMB* expression exhibited a 1.5 log-fold higher expression in ARLD patients in comparison to CVH patients (*p* = 0.01). *GZMK* and *TIGIT* were upregulated in HCC compared to ARLD (*p* = 0.03). Further analysis showed no substantial expression patterns of other exhaustion and memory genes, such as *CTLA4* and *HAVCR2,* among all groups. In contrast, expression of *LAG3* was upregulated in peri-tumor samples compared to *CVH* and ARLD groups (*p* = 0.02). These findings indicate an elevation of *LAG3* in the HCC microenvironment, highlighting its potential role in tumor immune evasion. Similarly, *PDCD1* was upregulated in the HCC group compared to CVH (*p* = 0.02), while there were no notable changes in the expression of this gene in the same group compared to ARLD (Supplementary Table S4).

Next, using a well-established single-cell RNA sequencing dataset by Ramachandran et al., we estimated hepatic cell type proportions from bulk RNA sequencing data. The single-cell deconvolution analysis did not reveal any major alternations in the relative proportions of immune and non-immune cell types across the groups (Supplementary Figure S2). The distribution of cell types populations such as T cells and B cells, remained consistent without notable variations between the conditions.

## 4 Discussion

Research over the past decades has shown that immune cell reprogramming plays a central role in the development of chronic liver diseases and hepatocellular carcinoma. Various etiologies including viral hepatitis, alcohol-related liver disease and metabolic dysfunction-associated steatotic liver disease trigger distinct inflammatory and fibrotic pathways involving several immune cells (Heymann and Tacke, 2016). A thorough understanding of immune regulation is crucial for grasping the pathogenesis of complex diseases and fibroinflammatory conditions as well as tumor microenvironments. To delineate immune cell reprogramming in ARLD, CVH, and viral hepatitis-related HCC, we performed combined bulk liver transcriptome as well as flow-cytometry-based immune cell characterization in PBMC and liver tissues.

Our study demonstrated that major peripheral and hepatic lymphocyte subgroups including NK, T and B cells were within the normal range, as previously defined by the other studies (Robinson et al., 2016; Bogdanos et al., 2013; Notas et al., 2009; Shi et al., 2011; Gao et al., 2008; Chen and Tian, 2021; Morsy et al., 2005). However, the most significant difference was observed in liver B cell frequency as ARLD indicating a lower count compared to other groups. This aligns with previous studies suggesting that chronic alcohol consumption leads to lower numbers of B cells. In contrast to relative B cell depletion in ARLD, studies suggested higher immunoglobulin production, particularly IgA (Chang et al., 2002; Wang et al., 2010; Patel et al., 2021; Aldo-Benson et al., 1992). Various hypotheses were proposed to explain the B cell depletion in excessive alcohol consumption; direct effect of alcohol on bone marrow suppression, acetaldehyde toxicity affecting NK cells, disrupted T cell cytokine balance, and compromised B cell traffic among the secondary lymphoid organs triggered by increased circulating lipopolysaccharides due to increased intestinal permeability are among the leading mechanisms identified (Patel et al., 2021; Almeida et al., 2015; Li et al., 2019; Matos et al., 2013).

Unaltered total hepatic CD3^+^ T cell counts but higher CD57^+^ T cell frequency in ARLD and CVH patients compared to HCC patients are the significant findings of this study. CD57 is associated with various biological functions such as senescence or differentiation (ElTanbouly and Noelle, 2021). In this context, it is likely that ARLD is associated with highly differentiated hepatic T cell populations compared to CVH and HCC. The supporting evidence for this proposal is the observation of *CD27* downregulation in ARLD compared to CVH and HCC. Given that T cells tend to lose co-stimulatory molecules such as CD27/28 and express CD57 to regulate inflammation with prolonged antigen exposure, ARLD-associated hepatic T cells may have induced into differentiated T cells towards effector memory T cells. As a result of this differentiation, T cells are expected to produce more cytotoxic cytokines such as granzyme and perforin (Bratke et al., 2005; Baars et al., 2005; Kared et al., 2014). Our results showed that the *GZMB* gene was upregulated in ARLD samples compared to CVH. This can be due to either the increased activation of T cells in ARLD or the relatively more T cell exhaustion in CVH.

CD27 expression is crucial not only for T cells but also for B cells, playing a vital role in determining class-switching. Classically, switched memory B cells are characterized by IgD^−^CD27^+^ and IgD^−^ CD27^−^ subsets, with the latter termed double-negative (Berkowska et al., 2011; Pieper et al., 2013). Specifically, within the double negative subsets, distinct populations of serum IgG^+^ and IgA^+^ class-switched memory B cells are delineated by CD27 expression, with a notable increase observed in ARLD (Wang et al., 2010; Matos et al., 2013). Additionally, another study highlighted a significant increase in the proportion of CD27^−^ serum IgA^+^ memory B lymphocytes among all serum IgA^+^ memory B cells in patients with ARLD compared to the control group (Almeida et al., 2015). Supporting this information, the downregulation of *CD27* in our results may be due to an increase in the class-switched memory B cells and plasma cells.

In this study, we noted a lower number of NK cell counts within peri-HCC hepatic tissues compared to those in ARLD-liver samples. This was evident not only in cell percentages but also in the function of the cells through the upregulation of the gene *TIGIT*, recognized as a prominent inhibitory marker for NK cells. The liver is notably abundant in NK cells. These cells play a pivotal role in tumor surveillance, and their inhibition has been associated with worsened prognosis/survival in human HCC patients (Hao et al., 2021; Eggert et al., 2016). Additionally, *TIGIT* serves as a significant exhaustion marker for T cells. Emerging studies highlight its immunosuppressive effects on CD8^+^ T cells via the CD155/*TIGIT* signaling pathway in hepatocellular carcinoma, indicating that *TIGIT*-expressing T cells exhibit compromised functioning against tumors (Zhang et al., 2020). Furthermore, we observed that *granzyme K* (*GZMK*), which is recognized as a hallmark of cellular immune aging, is upregulated in peri-tumor specimens. Functional experiments strongly suggest that *GZMK* ^+^ CD8^+^ T cells develop under the influence of an aging microenvironment and may contribute to an inflammatory phenotype through increased secretion of *GZMK* (Mogilenko et al., 2021; Bouwman et al., 2021). The analysis of differentially expressed genes from RNA sequence highlighted a substantial upregulation of *LAG3* in peritumor samples compared to other groups. The lymphocyte activation gene is a key inhibitor receptor playing a crucial role in immune responses, which is broadly expressed in immune cell types, including T cells, NK cells and other subsets, highlighting its importance in maintaining immune homeostasis and preventing it from activation (Couzin-Frankel, 2013). Studies have shown that tumor-infiltrating T cells exposed to tumor antigens lose their effector and tumor-killing functions. Over time, these cells express an increasing number and diversity of inhibitory receptors, such as *PDCD1* and *LAG3* (Triebel et al., 2006). Thus, *LAG3* upregulation in HCC may be due to its role in tumor-mediated immune escape and immune regulation. Chronic tumor antigen exposure in HCC leads to the exhaustion of tumor-infiltrating lymphocytes such as CD8+T cells. This upregulation of *LAG3* may limit effective anti-tumor immune response (Zhou et al., 2017; Arvanitakis et al., 2024). Programmed cell death protein-1, encoded by the *PDCD* gene, is another crucial immune checkpoint receptor expressed on the surface of activated cells such as B and T cells, playing a vital role in downregulating immune responses and promoting self-tolerance by inhibiting T cell inflammatory activity (Chen et al., 2023). In line with this view, there was a higher *PDCD1* expression in peri-tumor samples compared with CVH. By HCC development, it is likely that tumor-driven immune suppression and T cell exhaustion contribute to a further increase in *PDCD1* expression, making its upregulation more pronounced in this specific context.

This study was conducted with a small number of patients, limiting the generalizability of our findings across the broader population of CLD patients. Another limitation is the study’s static nature, which fails to capture the dynamic changes in gene expression that occur over the course of disease progression or in response to therapeutic interventions. We were not able to gather longitudinal data from the patients given the low possibility for patients to undergo resection or re-transplant. We attempted to isolate liver mononuclear cells from core liver biopsies, however, the yield was very low to allocate samples for flow cytometry and RNA sequencing. The samples analyzed through bulk RNA sequencing provided a comprehensive view of gene expression within liver tissues rather than single-cell sequencing, which may not represent cellular heterogeneity within hepatic parenchyma as well as averaging the expression signals across all cell types. Moreover, our findings were not supported by mechanistic studies such as cytokine assays or gene-specific editing. The lack of healthy control samples presents a constraint on our ability to draw comparative conclusions; however, we attempted to deconvolute our findings with comparative relatively healthy subjects from other studies. Another limitation is the study’s static nature, which fails to capture the dynamic changes in gene expression that occur over the course of disease progression or in response to therapeutic interventions.

## 5 Conclusion

Our study provides data derived from three distinct human liver diseases as ARLD, CVH and HCC, offering a direct and clinically relevant perspective that cannot be fully captured by *in vitro* experiments or animal models. By utilizing advanced methodologies such as RNA sequencing and flow cytometry, we comprehensively analyzed the cellular and molecular landscape of liver diseases. These approaches enable uncovering detailed insights into transcriptional profiles and immune dynamics associated with each clinical condition. This integrative analysis highlights the power of combining human-derived samples with technologies to advance our understanding of liver disease pathophysiology. In conclusion, our study addresses an important gap in the existing literature by providing a comparative analysis of the different etiologies. The intricate immune dynamics explored in our research revealed distinctive features between these three disease etiologies, shedding light on the nuanced complexities of chronic liver diseases.

## Data Availability

The data presented in the study are deposited in the GEO repository, accession number GSE287348.

## Abbreviations

HCC, Hepatocellular Carcinoma; CLD, Chronic Liver Disease; ARLD, Alcohol-Related Liver Disease; CVH, Chronic Viral Hepatitis; LN, Lineage; CD, Cluster of Differentiation; NK cells, natural killer cells; *TIGIT*, T cell immunoreceptor with Ig and ITIM domains; *KLRC1*, killer cell lectin like receptor C1; *GZMB*, granzyme B; *GZMK*, granzyme K; *CTLA4*, cytotoxic T-lymphocyte associated protein 4; *LAG3*, lymphocyte activating 3; *HAVCR2*, hepatitis A virus cellular receptor 2; *PDCD1*, programmed cell death 1.
