# Supplementary material for: Cellular and immune landscape of chronic liver diseases: insights from immunophenotyping
Source: Front Mol Biosci. 2025 Jan 29;11:1521811. doi: 10.3389/fmolb.2024.1521811 (PMC11813787; doi:10.3389/fmolb.2024.1521811)
Supplement: Supplementary file 1 [file DataSheet1.docx]

| **Brand** | **Antibody Name** | **Clone** | **Catalog Number** | **Dilution** | **BP Filter** | **Longpass Filter** | **Laser** |
| --- | --- | --- | --- | --- | --- | --- | --- |
| Biolegend | Brilliant Violet 421™ anti-human CD20 Antibody | 2H7 | 302329 | 1:200 | 440/50 | 417 | **405** |
|  | Brilliant Violet 605™ anti-human CD56 Antibody | HCD56 | 318333 | 1:200 | 603/48 | 555 |  |
|  | FITC anti-human CD57 Antibody | HNK-1 | 359603 | 1:200 | 530/30 | 495 | **488** |
|  | PE anti-human CD19 Antibody | HIB19 | 302208 | 1:200 | 590/40 | 555 |  |
|  | PE/Cyanine5 anti-human CD3 Antibody | HIT3a | 300310 | 1:200 | 695/40 | 650 |  |
|  | PE/Dazzle™ 594 anti-human CD4 Antibody | OKT4 | 317448 | 1:200 | 620/15 | 600 | **561** |
|  | PE/Cyanine7 anti-human CD45 Antibody | 2D1 | 368532 | 1:200 | 780/60 | 740 |  |
|  | Alexa Fluor® 700 anti-human CD8 Antibody | SK1 | 344723 | 1:200 | 720/30 | 690 | **640** |
|  | Zombie NIR™ Fixable Viability Kit | - | 423106 | 1:200 | 780/60 | 740 |  |

**Supplementary Table S1. Attune NxT Flow Cytometer Configuration and Antibody selection**

**Supplementary Table S2.Reagents and kits**

| **Brand** | **Name** | **Catalog Number** |
| --- | --- | --- |
| Biowest | Dulbecco’s Phosphate Buffered Saline | L0615 |
| Biowest | Fetal Bovine Serum | S160H |
| Sigma-Aldrich | Bovine Serum Albumin | A2153 |
| Serumwerk Bernburg | Ficoll (density 1.077 g/mL) | 1858 |
| PanReac Applichem | Dimethyl Sulfoxide | A3672,0100 |
| Zymo Research | Quick RNA Miniprep kit | R1054 |
| ThermoFisher Scientific | Qubit™ RNA High Sensitivity (HS), Broad Range (BR) kit | Q32852 |
| Illumina | Standard total RNA prep, Ribo-Zero Plus Kit | 20040525 |

**Supplementary Table S3. List of the selected genes for Bulk-RNA sequence analysis.** The analyzed genes were chosen from the literature for their relevance to the examined cell types complementary to the FACS results.

| **CD8^+^**  **T Cells** |  | **CD4^+^**  **T Cells** |  | **CD4^+^**  **Tregs** |  | **NK Cells** |  |
| --- | --- | --- | --- | --- | --- | --- | --- |
| **Gene** | **Ensemble ID** | **Gene** | **Ensemble ID** | **Gene** | **Ensemble ID** | **Gene** | **Ensemble ID** |
| ***GZMB*** | ENSG00000100453 | ***IL7R*** | ENSG00000168685 | ***FOXP3*** | ENSG00000049768 | ***GZMB*** | ENSG00000100453 |
| ***GZMK*** | ENSG00000113088 | ***CD38*** | ENSG00000004468 | ***CTLA4*** | ENSG00000163599 | ***GZMK*** | ENSG00000113088 |
| ***PRF1*** | ENSG00000180644 | ***HLA-DR*** | ENSG00000227993 | ***EBI3*** | ENSG00000105246 | ***PRF1*** | ENSG00000180644 |
| ***GNLY*** | ENSG00000115523 | ***CCR6*** | ENSG00000112486 | ***IL10*** | ENSG00000136634 | ***GNLY*** | ENSG00000115523 |
| ***GZMA*** | ENSG00000145649 | ***KLRB1*** | ENSG00000111796 | ***TGFB*** | ENSG00000105329 | ***GZMA*** | ENSG00000145649 |
| ***GZMH*** | ENSG00000100450 | ***CD27*** | ENSG00000139193 | ***IKZF2*** | ENSG00000030419 | ***GZMH*** | ENSG00000100450 |
| ***KLRG1*** | ENSG00000139187 | ***CCR7*** | ENSG00000126353 | ***IL2RA*** | ENSG00000134460 | ***KLRG1*** | ENSG00000139187 |
| ***IL7R*** | ENSG00000168685 | ***GZMB*** | ENSG00000100453 | ***NOTCH1*** | ENSG00000148400 | ***IFNG*** | ENSG00000111537 |
| ***IFNG*** | ENSG00000111537 | ***GZMK*** | ENSG00000113088 | ***NOTCH2*** | ENSG00000134250 | ***TNFA*** | ENSG00000228978 |
| ***CD52*** | ENSG00000169442 | ***PRF1*** | ENSG00000180644 | ***NOTCH3*** | ENSG00000074181 | ***CD38*** | ENSG00000004468 |
| ***B3GAT1*** | ENSG00000109956 | ***GNLY*** | ENSG00000115523 | ***NOTCH4*** | ENSG00000232339 | ***FCGR3A*** | ENSG00000203747 |
| ***CCR7*** | ENSG00000126353 | ***GZMA*** | ENSG00000145649 | ***TIGIT*** | ENSG00000181847 | ***B3GAT1*** | ENSG00000109956 |
| ***CCL5*** | ENSG00000271503 | ***GZMH*** | ENSG00000100450 | ***PDCD1*** | ENSG00000188389 | ***KLRC1*** | ENSG00000134545 |
| ***CRIP1*** | ENSG00000213145 | ***NKG7*** | ENSG00000105374 | ***HAVCR2*** | [ENSG00000135077](https://www.ensembl.org/Homo_sapiens/geneview?gene=ENSG00000135077) | ***KLRC2*** | ENSG00000205809 |
| ***CD74*** | ENSG00000019582 | ***CCL4*** | ENSG00000275824 | ***LAG3*** | ENSG00000089692 | ***KLRC3*** | ENSG00000205810 |
| ***CD69*** | ENSG00000110848 | ***CCL5*** | ENSG00000271503 |  |  | ***KLRK1*** | ENSG00000213809 |
| ***CD38*** | ENSG00000004468 | ***CD69*** | ENSG00000110848 |  |  | ***NCR1*** | ENSG00000276450 |
| ***KLRC1*** | ENSG00000134545 | ***B3GAT1*** | ENSG00000109956 |  |  | ***CSF2*** | ENSG00000164400 |
| ***KLRC2*** | ENSG00000205809 | ***IFNG*** | ENSG00000111537 |  |  | ***CD27*** | ENSG00000139193 |
| ***KLRC3*** | ENSG00000205810 | ***RORC*** | ENSG00000143365 |  |  | ***NKG7*** | ENSG00000105374 |
| ***KLRK1*** | ENSG00000213809 | ***PDCD1*** | ENSG00000188389 |  |  | ***HAVCR2*** | [ENSG00000135077](https://www.ensembl.org/Homo_sapiens/geneview?gene=ENSG00000135077) |
| ***PDCD1*** | ENSG00000188389 | ***CTLA4*** | ENSG00000163599 |  |  | ***LAG3*** | ENSG00000089692 |
| ***CTLA4*** | ENSG00000163599 | ***HAVCR2*** | [ENSG00000135077](https://www.ensembl.org/Homo_sapiens/geneview?gene=ENSG00000135077) |  |  |  |  |
| ***HAVCR2*** | [ENSG00000135077](https://www.ensembl.org/Homo_sapiens/geneview?gene=ENSG00000135077) | ***LAG3*** | ENSG00000089692 |  |  |  |  |
| ***LAG3*** | ENSG00000089692 |  |  |  |  |  |  |

**
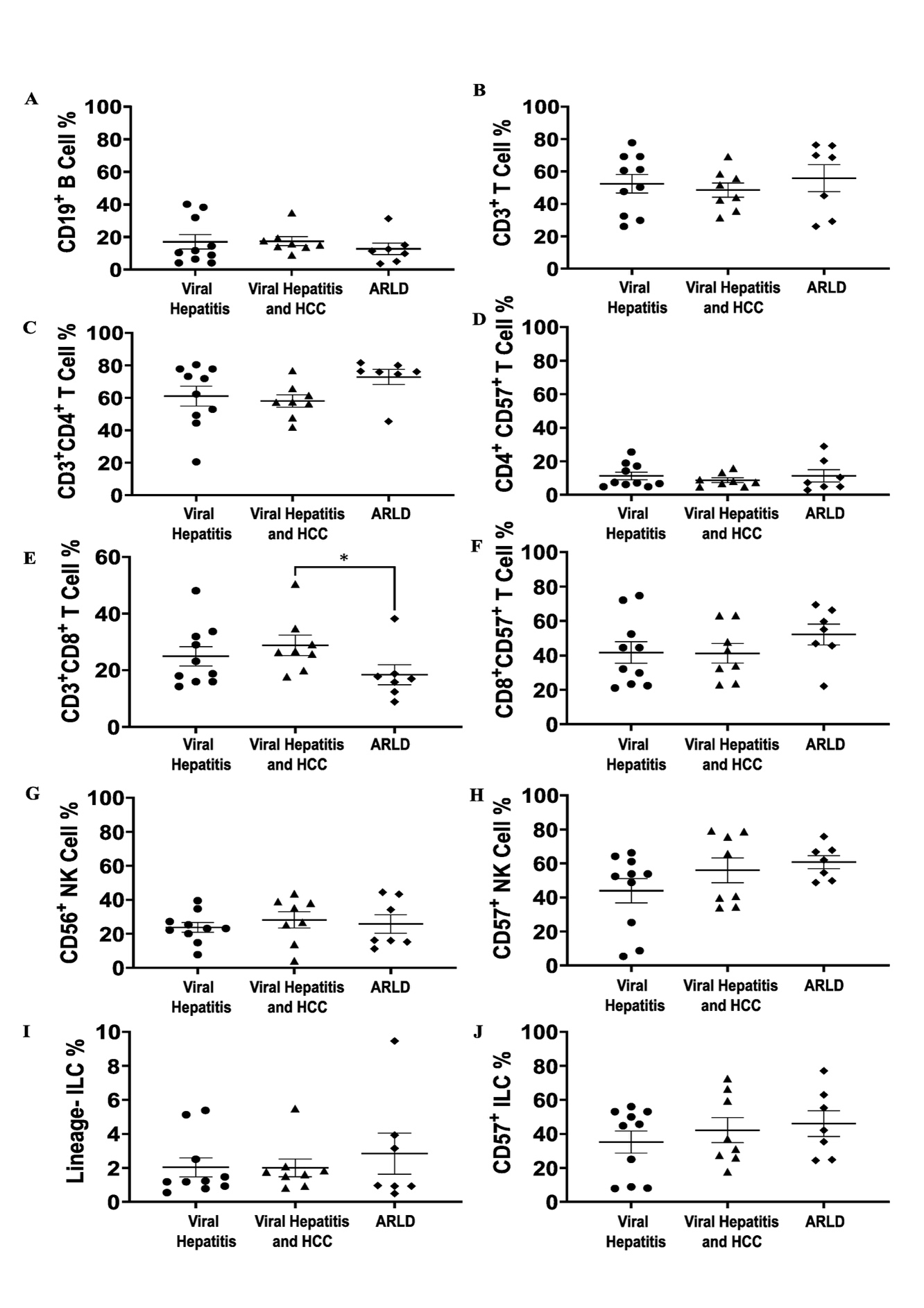
Supplementary Figure S1.Peripheral blood mononuclear cells immunophenotyping of different cohorts.** Shapiro-Wilk test was used for the normality assessment. Non-normally distributed variables were assessed by Kruskal-Wallis with Dunn’s Correction. * p<0.05. (A), (B) Total blood B and T lymphocyte proportion in three groups. (C), (D) CD4^+^ T helpers and CD4^+^CD57^+^ T helper contents. (E) CD3^+^CD8^+^ CTL showed lower level in ARLD compared to the other groups. (F) CD57^+^CD8^+^ CTL. (G), (H) Natural killer cells proportion. (I), (J) Blood lineage and CD57^+^ innate lymphocyte proportions in three etiologies.

**Supplementary Figure S2. Single-cell deconvolution analysis elucidates cellular heterogeneity and its correlation with chronic liver disease pathogenesis.** Shapiro-Wilk test was used for the normality assessment. Normally distributed data were analysed with One-Way ANOVA, and non-normally distributed variables were assessed using Kruskal-Wallis with Dunn’s post-hoc test.

**
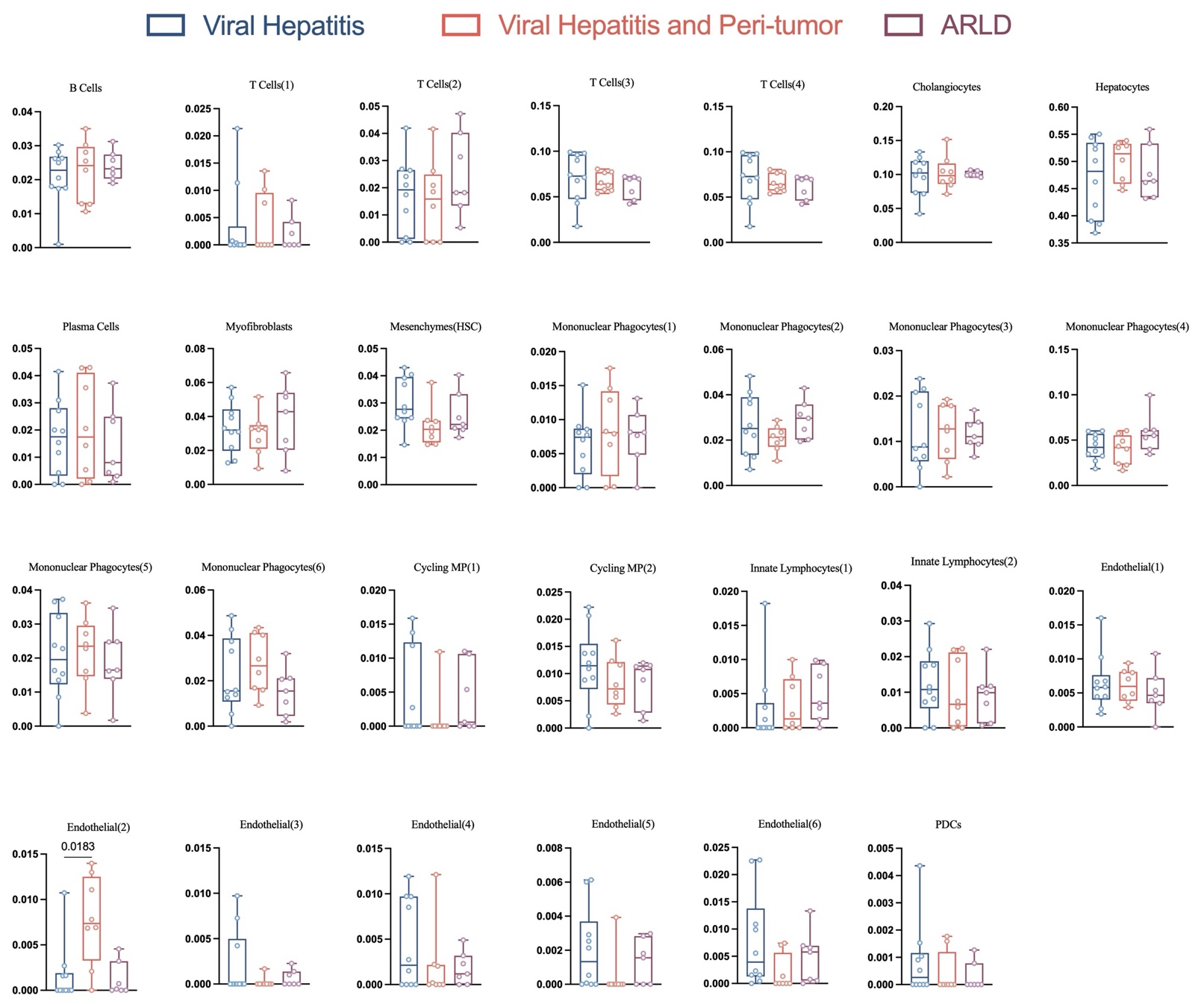
**

**Supplementary Table S4. Bulk RNA sequence data regarding the studied DEGs among three groups.**

|  |  | | **CVH with HCC vs ARLD** | | | |  | |  | |
| --- | --- | --- | --- | --- | --- | --- | --- | --- | --- | --- |
| **gene_names** | **baseMean** | | **log2FoldChange** | | **lfcSE** | | **stat** | | **pvalue** | |
| *CTLA4* | 53,2361589 | | 0,58609704 | | 0,67886412 | | 0,86334956 | | 0,38794531 | |
| *LAG3* | 73,9897734 | | 1,199907461 | | 0,51425013 | | 2,33331484 | | 0,01963163 | |
| *HAVCR2* | 280,868116 | | 0,099873931 | | 0,25974219 | | 0,38451177 | | 0,70059917 | |
| *PDCD1* | 50,7205276 | | 0,437644444 | | 0,6915146 | | 0,63287809 | | 0,52681326 | |
| *GZMK* | 88,9149276 | | 0,89557552 | | 0,41565201 | | 2,15462814 | | 0,03119095 | |
| *GZMB* | 20,4183696 | | -0,920035351 | | 0,67025792 | | -1,3726587 | | 0,16985848 | |
| *TIGIT* | 73,7611372 | | 0,923992343 | | 0,43895155 | | 2,10499849 | | 0,03529144 | |
| *KLRC1* | 38,6635789 | | -0,733207312 | | 0,3586296 | | -2,0444696 | | 0,04090718 | |
| *CD27* | 88,2787345 | | 2,105921672 | | 0,57812561 | | 3,64267149 | | 0,00026982 | |
|  |  | |  | |  | |  | |  | |
| **CVH with HCC vs CVH** | | | | | | | | | | |
| **gene_names** | | **baseMean** | | **log2FoldChange** | | **lfcSE** | | **stat** | | **pvalue** |
| *CTLA4* | | 53,2361589 | | 0,538237089 | | 0,62382843 | | 0,86279666 | | 0,38824928 |
| *LAG3* | | 73,9897734 | | 1,233800035 | | 0,55940352 | | 2,20556359 | | 0,02741457 |
| *HAVCR2* | | 280,868116 | | 0,302057079 | | 0,23918124 | | 1,26287947 | | 0,2066325 |
| *PDCD1* | | 50,7205276 | | 1,405820782 | | 0,63926109 | | 2,19913398 | | 0,0278684 |
| *GZMK* | | 88,9149276 | | 0,537294795 | | 0,38139399 | | 1,40876578 | | 0,15890444 |
| *GZMB* | | 20,4183696 | | 0,649067072 | | 0,62909196 | | 1,03175229 | | 0,30218818 |
| *IGIYT* | | 73,7611372 | | 0,491223553 | | 0,40250268 | | 1,22042305 | | 0,22230454 |
| *KLRC1* | | 38,6635789 | | -0,694533902 | | 0,33228196 | | -2,0901945 | | 0,03660034 |
| *CD27* | | 88,2787345 | | 0,78360268 | | 0,52700217 | | 1,48690598 | | 0,13703964 |

| **ARLD vs CVH** | | | | | |
| --- | --- | --- | --- | --- | --- |
| **gene_names** | **baseMean** | **log2FoldChange** | **lfcSE** | **stat** | **pvalue** |
| *CTLA4* | 53,2361589 | -0,047859952 | 0,64962296 | -0,0736734 | 0,94127024 |
| *LAG3* | 73,9897734 | -0,033892573 | 0,53702205 | -0,0631121 | 0,94967726 |
| *HAVCR2* | 280,868116 | 0,202183148 | 0,24861652 | 0,81323297 | 0,4160845 |
| *PDCD1* | 50,7205276 | 0,968176338 | 0,66479591 | 1,45635122 | 0,14529555 |
| *GZMK* | 88,9149276 | -0,358280725 | 0,39871811 | -0,8985815 | 0,36887561 |
| *GZMB* | 20,4183696 | 1,569102423 | 0,64791855 | 2,42175876 | 0,0154456 |
| *IGIYT* | 73,7611372 | -0,43276879 | 0,42117888 | -1,0275178 | 0,30417671 |
| *KLRC1* | 38,6635789 | 0,03867341 | 0,34006607 | 0,11372323 | 0,9094572 |
| *CD27* | 88,2787345 | -1,322318992 | 0,55334384 | -2,3896878 | 0,0168627 |
